# Early Neurological Improvement and Ambulation Recovery After Delayed Surgery in Surgically Selected Nonambulatory Metastatic Epidural Spinal Cord Compression: A Retrospective Cohort Study

**DOI:** 10.3390/curroncol33050299

**Published:** 2026-05-20

**Authors:** Aydin Talat Baydar, Baran Taskala, Bahadir Topal, Muhammed Bayindir, Yunus Emre Batman, Ilhan Yilmaz, Ali Dalgic

**Affiliations:** 1 Department of Neurosurgery, Basaksehir Cam and Sakura City Hospital, Istanbul 34480, Turkey; baran.taskala@saglik.gov.tr (B.T.); bahadir.topal@saglik.gov.tr (B.T.); muhammed.bayindir1@saglik.gov.tr (M.B.); yunusemre.batman@saglik.gov.tr (Y.E.B.); ilhan.yilmaz@sbu.edu.tr (I.Y.); 2 Department of Neurosurgery, Medicana International Hospital, Ankara 06510, Turkey; ali.dalgic@medicana.com.tr

**Keywords:** metastatic epidural spinal cord compression, delayed surgery, neurological recovery, ambulation recovery, spine oncology

## Abstract

Delayed decompression for metastatic epidural spinal cord compression (MESCC) is a common real-world problem, yet short-interval recovery after patients have already remained nonambulatory for at least 48 h is poorly defined. We retrospectively evaluated 41 surgically selected patients with MRI-confirmed epidural MESCC (Bilsky grade 2–3) and preoperative nonambulatory neurological deficit (Frankel grades A–C) lasting at least 48 h. The primary outcome was early neurological improvement, defined as a gain of at least one Frankel grade by postoperative days 10–14. The secondary outcome was early ambulation recovery, defined as postoperative Frankel grade D or E at the same interval. Early neurological improvement occurred in 20/41 patients (48.8%), and early ambulation recovery occurred in 15/41 (36.6%). No patient received postoperative index-level radiotherapy before the POD10–14 neurological assessment. Recovery was most common among patients with baseline Frankel grade C. In exploratory adjusted Firth-penalized models, ECOG performance status 3–4 was associated with lower odds of both outcomes. Rapid-growth tumors, classified using a pragmatic adapted growth-category framework, were associated with lower odds of early neurological improvement. Baseline Frankel grade C favored early ambulation recovery. Higher standardized HALP showed an exploratory association with early neurological improvement but did not alter the main clinical interpretation. Meaningful early recovery was observed in a subset of surgically selected MESCC patients despite delayed surgery, although these findings do not establish equivalence to earlier surgery or isolate the effect of surgery from multimodal oncologic care.

## Simple Summary

Metastatic epidural spinal cord compression (MESCC) is an oncologic emergency that can cause profound weakness, paralysis, and loss of ambulation. This study focused on a specific real-world scenario that remains incompletely defined: surgically selected patients who had already remained nonambulatory for at least 48 h before decompression. Among 41 such patients, nearly half showed early neurological improvement, and more than one-third achieved early ambulation recovery within postoperative days 10–14. Poorer performance status was associated with worse outcomes, whereas preserved preoperative motor function favored ambulation recovery. Tumors classified within a pragmatic adapted growth-category framework showed lower recovery rates in the rapid-growth group. HALP showed only a secondary exploratory association with early neurological improvement and should be viewed as adjunctive context rather than a decision-defining variable. No patient received postoperative index-level radiotherapy before the POD10–14 neurological assessment. These findings suggest that meaningful early recovery may still occur in carefully selected patients despite delayed surgery, although recovery remains strongly conditioned by baseline neurological and systemic status.

## 1. Introduction

Metastatic epidural spinal cord compression (MESCC) is one of the most time-sensitive complications of systemic cancer because progressive spinal cord compromise can rapidly lead to irreversible neurological loss, loss of ambulation, pain, and reduced quality of life [1,2]. In selected patients, surgical decompression with or without stabilization remains a major component of management, particularly in the presence of high-grade epidural compression, mechanical instability, or progressive neurological deterioration within a multidisciplinary oncologic framework [1,3].

Although early intervention is generally preferred, real-world care pathways are frequently less ideal. Delays may arise from late presentation, diagnostic uncertainty, referral inefficiencies, medical optimization, evolving oncologic planning, or patient-related decision delay [4,5,6]. As a result, some patients undergo surgery only after they have already been nonambulatory for 48 h or longer. Once this threshold has been crossed, the clinically relevant question changes: rather than whether earlier decompression would have been preferable, the immediate issue becomes what degree of neurological and functional recovery may still remain achievable.

Published work suggests that recovery after delayed surgery is heterogeneous and likely shaped by baseline neurological status, systemic condition, and tumor-related factors [7,8,9,10]. However, practical counseling remains difficult because many studies focus primarily on timing thresholds, while providing limited detail regarding patient-level recovery trajectories once delay has already occurred. In particular, clinicians and families need realistic information on how often early improvement occurs after prolonged nonambulatory deficit and how recovery differs according to preoperative neurological grade and broader oncologic context.

Host reserve may also influence recovery after decompression. The hemoglobin–albumin–lymphocyte–platelet (HALP) score is a composite index integrating parameters related to oxygen-carrying capacity, nutritional state, immune competence, and inflammatory burden, and it has been associated with prognosis across multiple malignancies [11,12,13,14]. In MESCC, however, HALP should be regarded as an exploratory contextual marker rather than as a validated clinical decision variable, particularly in retrospective surgical cohorts where corticosteroid exposure, acute illness, and cancer burden may substantially influence laboratory values.

This study was designed as a recovery-after-delay analysis rather than a timing-comparison study. We evaluated surgically selected nonambulatory MESCC patients who had already crossed the at least 48 h deficit threshold and characterized early postoperative day (POD) 10–14 neurological improvement and ambulation recovery. The objective was not to determine whether delayed surgery is equivalent to earlier surgery, nor to isolate the independent effect of surgery from other oncologic treatments, but to describe early recovery patterns and clinically relevant correlates in this difficult real-world subgroup. To better contextualize operative selection and early recovery patterns, we also characterized delay reasons, perioperative risk status, known-primary status, vertebral metastatic extent, and surgical target location in this surgically selected delayed-MESCC cohort.

## 2. Materials and Methods

### 2.1. Study Design and Patient Selection

This retrospective cohort study was conducted at a tertiary spine oncology center in accordance with the Declaration of Helsinki and approved by the institutional ethics committee (Approval No: 15.10.2025-369). The requirement for informed consent was waived because of the retrospective design and use of anonymized data. This study was reported in accordance with the STROBE statement for cohort studies.

Consecutive patients undergoing surgery for spinal metastases between June 2020 and September 2025 were screened. No a priori sample size calculation was performed because this was a retrospective exploratory cohort study; all consecutive eligible surgically treated patients during the study period were included. Inclusion criteria were: (1) MRI-confirmed epidural spinal cord compression (Bilsky grade 2–3) [15]; and (2) nonambulatory neurological deficit lasting at least 48 h preoperatively (Frankel grades A–C). Exclusion criteria were age <18 years, primary or intradural tumors, prior surgery at the index level, or absence of early postoperative neurological assessment at postoperative days (POD) 10–14.

Surgical candidacy was determined through multidisciplinary assessment using NOMS principles [3], and the cohort therefore reflects surgically selected patients judged appropriate for decompression in the context of advanced systemic cancer.

### 2.2. Surgical Treatment

Surgical treatment consisted of posterior decompression alone, decompression with stabilization, or corpectomy-based reconstruction according to compression pattern, mechanical stability, oncologic context, and overall condition. Most operations, including decompression with stabilization and corpectomy-based reconstruction, incorporated a separation-surgery principle: decompression of the thecal sac and creation of a margin between epidural tumor and neural elements when safely achievable. A minority of decompression-only procedures were intentionally limited to posterior decompression without formal circumferential separation because of patient condition, surgical goal, or anatomical constraints. The need for stabilization was guided by the Spinal Instability Neoplastic Score (SINS) [16], with instrumentation added when instability or impending instability was present. All procedures were performed by, or under the direct supervision of, a surgeon with at least five years of dedicated spine oncology experience.

For lymphoma and other radiosensitive tumors, surgery was reserved for progressive neurological deficit despite or prior to systemic or radiation treatment, diagnostic necessity in cases without established tissue diagnosis, or mechanical instability, in keeping with NOMS-based decision-making for high-grade cord compression [3]. In contemporary practice, separation surgery followed by high-dose stereotactic radiotherapy may provide durable local control in selected patients; however, the present analysis focused on early neurological recovery after decompression rather than on longer-term local control or radiotherapy sequencing.

### 2.3. Clinical and Laboratory Variables

Baseline neurological status was graded using the Frankel classification. Baseline Frankel grade for all analyses was defined as the neurological grade documented immediately before surgery by the treating neurosurgical team. Duration of nonambulatory deficit was calculated from the first documented inability to ambulate to the date of surgery. Epidural compression severity was assessed using the Bilsky scale [15], performance status using the Eastern Cooperative Oncology Group (ECOG) scale [17], and frailty using the Metastatic Spinal Tumor Frailty Index (MSTFI) [18].

Primary tumors were grouped using an adapted Katagiri-style growth-category framework to provide a pragmatic estimate of broad biologic tempo [19]. Because molecular treatment status, hormone sensitivity, and other disease-specific modifiers were not consistently available, this classification was not intended to reproduce the original Katagiri prognostic score. For regression analyses, tumors were dichotomized as rapid-growth versus non-rapid-growth. The distribution of individual primary tumor subtypes across these adapted categories is provided in Supplementary Table S1.

Preoperative laboratory values were obtained within five days before surgery. HALP was calculated as hemoglobin × albumin × lymphocyte count/platelet count [11,20]. For regression analyses, HALP was standardized as a z-score (HALP^z^) by subtracting the cohort mean and dividing by the cohort standard deviation; effect estimates therefore represent a 1-standard-deviation increase in HALP. Because albumin and lymphocyte counts may be influenced by corticosteroid exposure, acute illness, systemic inflammation, and overall cancer burden, HALP was treated as an exploratory adjunctive variable reflecting host reserve rather than as a stand-alone prognostic or decision-making tool.

Perioperative oncologic treatment data were reviewed from institutional oncology notes, radiation oncology records, discharge summaries, and available external treatment documentation. Radiotherapy variables included preoperative radiotherapy to the index spinal level, any preoperative radiotherapy, postoperative radiotherapy to the index spinal level before the POD10–14 neurological assessment, any documented postoperative radiotherapy, and available radiotherapy details including treatment field, fractionation, and dose when recorded. Systemic therapy status was also reviewed descriptively when available. Because many patients received oncologic care across external centers and treatment records were heterogeneous, radiotherapy and systemic therapy variables were summarized descriptively and were not included in multivariable models.

### 2.4. Delay Classification

Delay was defined as a clinically documented interval during which decompression was not performed despite established MESCC with progressive neurological deficit, attributable to patient-, system-, or treatment-related factors. Delay reason was classified into six mutually exclusive categories using a prespecified prioritization hierarchy: (1) system access/referral delay; (2) medical optimization; (3) oncologic sequencing/multidisciplinary planning; (4) patient decision/consent; (5) intercurrent event; and (6) unknown/undocumented delay.

Adjudication was performed by retrospective review of admission notes, oncology documentation, multidisciplinary consultations, anesthetic records, and operative planning notes. Because classification was performed by a single senior reviewer without formal inter-rater reliability assessment, delay taxonomy findings were considered descriptive.

### 2.5. Outcomes

The primary outcome was early neurological improvement, defined as an increase of at least one Frankel grade from the immediately preoperative status by POD10–14. The secondary outcome was early ambulation recovery, defined as achievement of Frankel grade D or E at the same interval. This early assessment window was chosen to capture short-interval neurological change after decompression before longer-term rehabilitation, radiotherapy, and systemic therapy effects could be reliably assessed [8]. Accordingly, the endpoints should be interpreted as early postoperative recovery outcomes rather than durable oncologic or functional endpoints.

The timing of postoperative index-level radiotherapy relative to this early neurological assessment was specifically recorded to evaluate whether early recovery preceded or followed postoperative radiotherapy exposure.

Recovery trajectories were additionally characterized using a preoperative-to-postoperative Frankel transition matrix.

### 2.6. Statistical Analysis

Analyses were performed in R v4.5.2. Categorical variables are reported as frequencies and percentages, and continuous variables as median (IQR). Unadjusted comparisons used Fisher’s exact test for categorical variables and the Mann–Whitney U test for continuous variables. To reduce sparse-data bias in multivariable analysis, associations were estimated using Firth-penalized logistic regression with profile-likelihood 95% confidence intervals; to reduce outcome heterogeneity, endpoints were defined at POD10–14. This approach was preferred because conventional logistic regression showed quasi-complete separation for ECOG performance status.

Given the limited sample size and low number of outcome events, model construction was intentionally parsimonious and based on a small set of clinically relevant variables selected a priori for their bedside interpretability and potential confounding relevance: baseline neurological status, overall performance status, and tumor growth category. HALP^z^ was then added in a secondary exploratory model to examine whether an adjunctive host-factor measure materially changed the clinical associations. The modeling strategy was therefore designed for cautious contextual estimation rather than for prediction-model development. Two adjusted models were constructed for each endpoint. There were no missing data for the primary outcome, secondary outcome, or covariates included in the adjusted models; therefore, complete-case analysis was performed. Model A included baseline Frankel grade (C vs. A–B), ECOG performance status (3–4 vs. 0–2), and tumor growth category (rapid-growth vs. non-rapid-growth). Model B added HALP^z^ as a secondary exploratory covariate. No formal discrimination or internal-validation analyses were performed because the models were intended to provide cautious exploratory association estimates rather than predictive performance metrics.

Radiotherapy and systemic therapy variables were analyzed descriptively because of small sample size, incomplete external treatment documentation, and heterogeneity of radiotherapy fields and regimens. These variables were not added to the Firth-penalized models to avoid overfitting and misclassification-driven inference.

The final events-per-variable ratios were 5.0 for the primary endpoint and 3.75 for the secondary endpoint; therefore, all adjusted estimates were considered exploratory and hypothesis-generating. All tests were two-sided, and *p* < 0.05 was considered statistically significant.

## 3. Results

### 3.1. Cohort Characteristics

**Figure 1:**
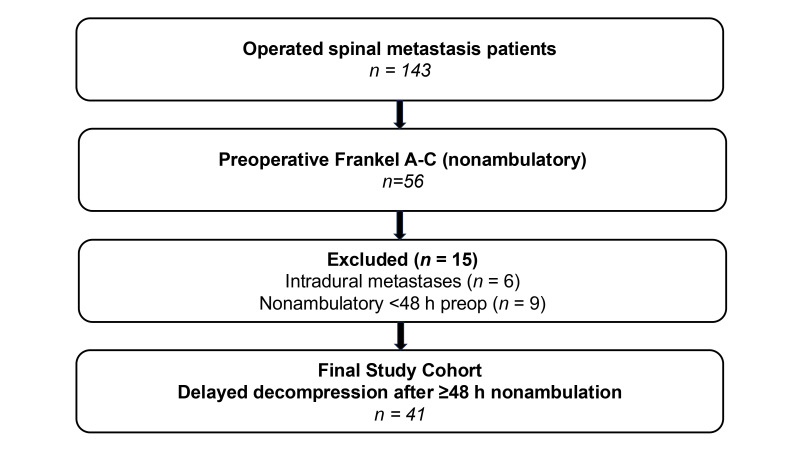
Study flow diagram of cohort selection for delayed decompression in nonambulatory MESCC. Consecutive operated spinal metastasis patients were screened for eligibility. The final cohort included patients with preoperative nonambulatory neurological deficit (Frankel grades A–C) lasting at least 48 h before surgery. Exclusions comprised intradural metastases and patients with nonambulatory deficit duration <48 h. MESCC, metastatic epidural spinal cord compression; h, hours.

During the study period, 143 patients underwent surgery for spinal metastases. Of these, 56 had preoperative Frankel A–C neurological status. Intradural metastases (*n* = 6) and patients with Frankel A–C deficit duration <48 h (*n* = 9) were excluded, yielding a final analytic cohort of 41 patients (Figure 1). The cohort was predominantly male (*n* = 33, 80.5%) with a median age of 65.0 years (IQR 58-71). All patients were nonambulatory at presentation: 4 (9.8%) had Frankel grade A, 14 (34.1%) Frankel grade B, and 23 (56.1%) Frankel grade C. Bilsky grade 3 compression was present in 31 patients (75.6%) and grade 2 compression in 10 (24.4%). Median SINS was 10 (IQR 8–12), and only 6 patients (14.6%) were categorized as mechanically stable. The majority of patients had ECOG performance status 3–4 (*n* = 36, 87.8%).

The most common primary tumor subtypes are summarized in Supplementary Table S1; under the pragmatic adapted growth-category framework, 14 cases (34.1%) were classified as rapid-growth and 27 (65.9%) as non-rapid-growth. Baseline characteristics and unadjusted comparisons according to early neurological improvement are shown in Table 1. Corresponding baseline comparisons according to early ambulation recovery are provided in Supplementary Table S2.

### 3.2. Disease Burden, Known-Primary Status, ASA Class, and Surgical Target Location

Primary tumor was known preoperatively in 20 patients (48.8%) and unknown in 21 (51.2%). ASA physical status was II in 9 patients (22.0%), III in 26 (63.4%), and IV in 6 (14.6%); an emergency modifier was attached to the ASA score in 14 patients (34.1%). Multivertebral metastatic involvement was common: 36 patients (87.8%) had two or more involved vertebrae, and 22 (53.7%) had more than four involved vertebrae. The maximal-compression surgical target was most commonly within the thoracic semirigid segment (T3–T10; *n* = 23, 56.1%), followed by the thoracolumbar junction (T11–L1; *n* = 7, 17.1%) and lumbar mobile segment (L2–L4; *n* = 5, 12.2%).

### 3.3. Perioperative Oncologic Treatment Context

Perioperative oncologic treatment data are summarized in Table 2. Preoperative radiotherapy to the index spinal level was documented in 6 patients (14.6%), and any preoperative radiotherapy was documented in 10 patients (24.4%). No patient received postoperative index-level radiotherapy before the POD10–14 neurological assessment. Any postoperative radiotherapy was documented in 14 patients (34.1%), whereas no postoperative radiotherapy was documented in 12 patients (29.3%) and postoperative radiotherapy status remained unknown in 15 patients (36.6%). Available radiotherapy details were heterogeneous and included palliative conventional external-beam radiotherapy, volumetric modulated arc therapy (VMAT)/intensity-modulated radiotherapy (IMRT)-based treatment, or stereotactic radiosurgery depending on field and indication. Systemic therapy regimen information was documented in 19 patients (46.3%), no systemic therapy was documented in 11 (26.8%), and systemic therapy status was unknown in 11 (26.8%). Because systemic treatment timing and continuity were heterogeneous across external oncology centers, these data were considered descriptive only.

### 3.4. Delay Characteristics and Duration

Referral/access delay (*n* = 12, 29.3%) and patient decision/consent delay (*n* = 10, 24.4%) were the most common primary delay categories, followed by oncologic sequencing/multidisciplinary planning (*n* = 8, 19.5%), medical optimization (*n* = 5, 12.2%), unknown/undocumented delay (*n* = 5, 12.2%), and intercurrent event delay (*n* = 1, 2.4%). Delay category was not associated with early neurological improvement (*p* = 0.819) or ambulation recovery (*p* = 0.607).

Median preoperative nonambulatory deficit duration was 7.0 days (IQR 3.0–12.0; range 2–30 days). Deficit duration did not differ between patients with and without early neurological improvement [7.5 (IQR 4.8–10.2) vs. 7.0 (IQR 3.0–14.0) days; *p* = 0.753] or between those who did and did not regain ambulation [8.0 (IQR 5.0–13.0) vs. 7.0 (IQR 3.0–11.5) days; *p* = 0.414].

### 3.5. Early Neurological and Ambulation Recovery

Early neurological improvement occurred in 20 of 41 patients (48.8%) by POD10–14. Early ambulation recovery to Frankel grade D or E occurred in 15 of 41 patients (36.6%).

### 3.6. Frankel Transition Analysis

**Figure 2:**
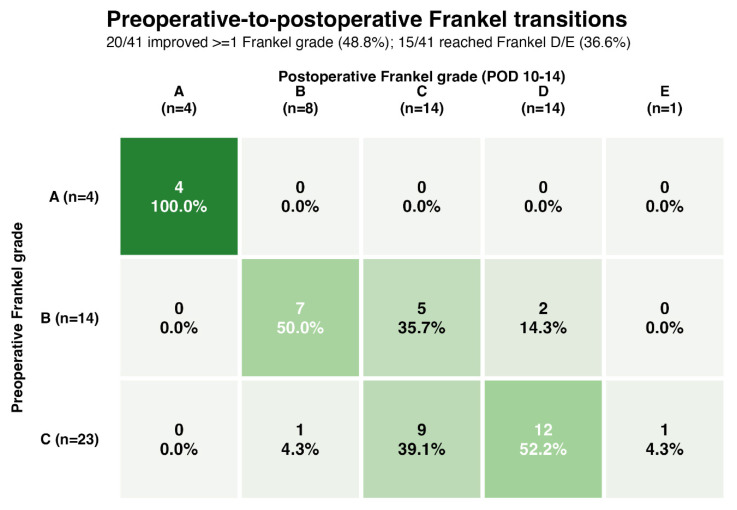
Heatmap of preoperative-to-postoperative Frankel transitions at POD10–14. Each cell shows the number of patients and the percentage within each preoperative Frankel category. Overall, 20/41 patients improved by at least one Frankel grade and 15/41 reached postoperative Frankel grade D or E. Darker shading indicates a greater proportion within the corresponding preoperative stratum. POD, postoperative day.

Preoperative-to-postoperative Frankel transitions are shown in Figure 2. None of the four patients presenting with Frankel grade A achieved early neurological improvement or early ambulation recovery. Among 14 patients with baseline Frankel grade B, 7 (50.0%) improved by at least one Frankel grade, and 2 (14.3%) reached the ambulatory zone by POD10–14. Among 23 patients with baseline Frankel grade C, 13 (56.5%) improved by at least one grade and 13 (56.5%) reached Frankel D or E, including one patient who achieved Frankel grade E.

### 3.7. Primary Endpoint: Early Neurological Improvement

In unadjusted analyses, ECOG performance status 3–4 (75.0% among improved vs. 100.0% among non-improved; *p* = 0.008), lower albumin (*p* = 0.042), and lower HALP (*p* = 0.017) were associated with absence of early neurological improvement. Rapid-growth tumors were more frequent among non-improved patients than among improved patients (47.6% vs. 20.0%; *p* = 0.100). All 5 patients with ECOG 0–2 showed neurological improvement, compared with 15 of 36 patients (41.6%) with ECOG 3–4.

In Model A, ECOG 3–4 (OR 0.049, 95% CI 0.000–0.640; *p* = 0.018) and rapid-growth tumors within the adapted growth-category framework (OR 0.215, 95% CI 0.036–0.916; *p* = 0.037) were associated with lower odds of early neurological improvement, whereas baseline Frankel grade C versus A–B was not significant (OR 1.270, 95% CI 0.325–5.091; *p* = 0.730). After addition of HALPz in Model B, higher standardized HALP showed an exploratory association with early neurological improvement (OR 6.292, 95% CI 1.226–61.424; *p* = 0.017), ECOG 3–4 remained unfavorable (OR 0.037, 95% CI 0.000–0.492; *p* = 0.009), and the rapid-growth association attenuated to borderline significance (OR 0.206, 95% CI 0.026–1.030; *p* = 0.055). Adjusted Firth-penalized logistic regression results for early neurological improvement are presented in Table 3.

### 3.8. Secondary Endpoint: Ambulation Recovery

In Model A, baseline Frankel grade C (vs. A–B) was associated with early ambulation recovery (OR 6.205, 95% CI 1.276–41.679; *p* = 0.023). ECOG 3–4 was associated with lower odds of early ambulation recovery (OR 0.046, 95% CI 0.000–0.632; *p* = 0.017), whereas rapid-growth tumors showed an unfavorable direction that did not meet conventional significance (OR 0.179, 95% CI 0.016–1.048; *p* = 0.057). All 5 patients with ECOG 0–2 regained ambulation, compared with 10 of 36 patients (27.8%) with ECOG 3–4. After HALPz addition in Model B, the main clinical pattern was unchanged: the association for Frankel grade C was attenuated (OR 3.585, 95% CI 0.614–26.712; *p* = 0.159), ECOG 3–4 remained unfavorable (OR 0.052, 95% CI 0.000–0.598; *p* = 0.014), and neither rapid-growth tumors (OR 0.253, 95% CI 0.024–1.473; *p* = 0.132) nor HALPz (OR 1.902, 95% CI 0.796–12.140; *p* = 0.198) was associated with early ambulation recovery. Adjusted Firth-penalized logistic regression results for early ambulation recovery are presented in Table 3.

### 3.9. Postoperative Course

Median postoperative length of stay was 6 days (IQR 3–8) overall and was shorter among patients who improved neurologically [4 days (IQR 3–6)] or regained ambulation [4 days (IQR 3–6)] than among those who did not [7 days (IQR 5–13) and 7 days (IQR 4–12), respectively]. Four patients (9.8%) underwent reoperation within 30 days, and all reoperations were wound-related.

## 4. Discussion

This study addresses a common but incompletely characterized clinical scenario: early recovery after decompression in patients with MESCC who have already remained nonambulatory for at least 48 h. In this surgically selected cohort, nearly half of patients showed early neurological improvement and more than one-third regained ambulation within 10–14 days. These findings do not challenge the principle that earlier decompression remains preferable [1,4,5,6]. Rather, they indicate that meaningful early recovery may still occur in selected patients even after the 48 h threshold has already been crossed. The results apply only to selected patients judged appropriate for surgery and should not be generalized to all delayed MESCC patients.

Among the clinical variables examined, performance status and tumor biologic tempo provided the clearest context for recovery after delay. ECOG 3–4 was unfavorable for both neurological improvement and ambulation recovery across both models. In absolute terms, neurological improvement occurred in all 5 patients with ECOG 0–2 but in only 15 of 36 patients with ECOG 3–4; ambulation recovery occurred in all 5 patients with ECOG 0–2 but in only 10 of 36 patients with ECOG 3–4. Rapid-growth tumors also showed an unfavorable direction, with an independent association for early neurological improvement and a similar but less stable pattern for ambulation recovery. From an oncology perspective, this remains plausible. ECOG status captures more than neurological deficit alone; it also reflects overall physiological reserve, disease burden, treatment tolerance, and the broader systemic condition in which decompression is undertaken [21]. Likewise, tumor biologic tempo may influence both local spinal progression and overall systemic trajectory. At the same time, the growth-category variable in this study was a pragmatic adapted Katagiri-style framework rather than a formal reconstruction of the original score, so these tumor-category findings should be interpreted cautiously [19].

Additional variables further contextualize the cohort as a high-burden surgically selected population. More than half of patients had more than four involved vertebrae, and nearly 90% had multivertebral metastatic involvement. Primary tumor status was unknown preoperatively in approximately half of the cohort, underscoring the diagnostic and oncologic uncertainty that often accompanies urgent MESCC surgery. ASA class was predominantly III or IV, supporting the interpretation that recovery after delay occurred within a medically vulnerable population rather than in an optimally selected elective surgical cohort.

Baseline neurological status remained highly relevant, particularly for functional recovery. Patients with Frankel grade C at presentation had the highest probability of early ambulation recovery, whereas no patient with Frankel grade A regained ambulation by POD10–14. Recovery to the ambulatory zone was concentrated in patients with preserved residual motor function, although two patients with baseline Frankel grade B did reach Frankel grade D at early follow-up. This pattern is clinically important because it provides a more realistic basis for bedside counseling than a simple binary statement that surgery is either worthwhile or futile after delay. The present findings are also consistent with recent delayed-MESCC literature showing that preserved baseline neurological function remains one of the most important correlates of postoperative ambulation recovery [7,8,9,10].

Our findings can also be interpreted alongside the recent delayed-MESCC study by Wänman et al., who examined patients who had lost walking ability for more than 48 h and reported restored ambulation in 61 of 111 evaluable patients (55%) at 1 month after surgery [7]. In our cohort, early ambulation recovery was observed in 15 of 41 patients (36.6%) at POD10–14. These rates are not directly comparable because the outcome windows differed and our analysis intentionally focused on an earlier postoperative interval, but both studies support the clinically relevant point that recovery after delayed decompression is heterogeneous rather than uniformly absent. Notably, Wänman et al. classified primary tumor grade according to Tomita et al. [22], which uses broad organ-based growth labels, whereas we used a pragmatic adapted Katagiri-style grouping to place tumor biology within a somewhat more contemporary prognostic framework. We do not present our approach as a formal replication of Katagiri scoring, because key disease-specific modifiers were unavailable retrospectively; however, the convergence of both studies toward worse recovery in more aggressive tumor categories supports the broader clinical point that biologic tempo remains relevant even after the 48 h threshold has already been crossed.

The surgical strategy should also be interpreted within contemporary spine oncology practice. Separation surgery combined with postoperative high-dose radiotherapy can provide more durable local control in appropriately selected patients, and this principle informed most decompression-with-stabilization and corpectomy-based procedures in the present cohort. Nevertheless, the current endpoints were early neurological improvement and ambulation recovery at POD10–14; therefore, the study does not evaluate durability of local control, radiotherapy response, or long-term oncologic outcomes.

HALP integrates hemoglobin, albumin, lymphocyte count, and platelet count, and has been linked to prognosis across multiple malignancies [11,12,13,14]. However, HALP added only limited exploratory context to the clinical model. Although higher HALP showed an association with early neurological improvement, this signal should be interpreted cautiously and should not displace the more clinically grounded variables in this study, namely baseline neurological status, performance status, and broad tumor biologic category. HALP integrates hemoglobin, albumin, lymphocyte count, and platelet count, but in MESCC these components are readily influenced by corticosteroid exposure, acute inflammatory state, nutritional depletion, anemia of chronic disease, hepatic function, and overall cancer burden. Accordingly, the present data do not support HALP as a stand-alone decision tool; at most, it may provide limited adjunctive host-reserve context in hypothesis-generating analyses.

Preoperative nonambulatory deficit duration was not associated with either endpoint within this already delayed cohort. This should not be interpreted as evidence that timing is irrelevant. Instead, once patients have already crossed a clinically unfavorable threshold of delay, other patient-specific factors, particularly residual motor function, systemic reserve, and tumor biologic tempo, may become more discriminative than the exact number of delayed hours [4,5,6,7]. In that sense, the present study is best understood as a recovery-after-delay study rather than a timing-comparison study.

This study was not designed to compare early versus delayed surgery. Therefore, the findings should not be interpreted as evidence that delayed surgery is equivalent to surgery within 48 h. Rather, the analysis focuses on patients who had already crossed the at least 48 h nonambulatory threshold, a scenario in which clinicians still need realistic information regarding residual recovery potential.

Radiotherapy is central to MESCC management and is particularly relevant when decompression is performed according to a separation-surgery principle. The most important finding for interpretation of the early endpoint was that no patient received postoperative index-level radiotherapy before the POD10–14 neurological assessment. Thus, the early neurological and ambulation recovery outcomes were assessed before postoperative index-level radiotherapy could plausibly account for the observed early recovery. Nevertheless, 6 patients had received preoperative index-level radiotherapy, 10 had received some form of preoperative radiotherapy, and postoperative radiotherapy was documented later in a subset of patients. Therefore, these data should not be interpreted as surgery-alone efficacy; rather, they describe early postoperative recovery patterns within a multimodal oncology population.

The distribution of delay reasons is also clinically informative. Referral/access barriers and patient decision/consent delays together accounted for more than half of cases. Although delay category itself was not associated with outcome in this small cohort, these patterns suggest that some pathways to delayed presentation may be modifiable through earlier referral, clearer communication, and better coordination across institutions and specialties.

Several limitations should be acknowledged. This was a single-center retrospective cohort of surgically selected patients, introducing selection bias and limiting generalizability to broader delayed-MESCC populations. The sample size was small, event counts were limited, and all adjusted estimates should be interpreted as exploratory. Although perioperative radiotherapy data were retrospectively reviewed and summarized, treatment documentation remained heterogeneous, particularly for patients treated across external centers. Postoperative index-level radiotherapy before POD10–14 was absent in this cohort, which supports the interpretability of the early neurological endpoint; however, preoperative radiotherapy exposure, later postoperative radiotherapy, incomplete external treatment details, and heterogeneous systemic therapy remain important limitations. The study therefore cannot determine the longer-term effect of radiotherapy dose, fractionation, field design, or systemic therapy sequencing on neurological durability, local control, or survival. In addition, the absence of a contemporaneous less than 48 h surgical comparator prevents conclusions regarding the relative effect of early versus delayed decompression. Steroid exposure was not consistently available for modeling and may have confounded both laboratory values and recovery. Tumor growth category was assigned using an adapted Katagiri-style framework rather than the full original schema, so some misclassification is possible. Outcomes were assessed early at POD10–14 and do not capture longer-term neurological, functional, or oncologic trajectories. Finally, delay taxonomy was adjudicated retrospectively by a single reviewer without formal reliability testing.

Overall, these data support a more nuanced view of delayed MESCC surgery. When patients present after prolonged nonambulatory deficit, the relevant question is not simply whether delay has occurred, but whether meaningful recovery may still remain achievable in the context of residual motor function, performance status, surgical candidacy, and tumor aggressiveness. Under that framing, early recovery after delayed surgery may still be observed in carefully selected patients, but the present data do not establish equivalence to earlier surgery or isolate the independent effect of surgery from multimodal oncologic care.

## 5. Conclusions

In this retrospective cohort of surgically selected patients with nonambulatory MESCC of at least 48 h duration, early neurological improvement occurred in nearly half of patients and early ambulation recovery in more than one-third. No patient received postoperative index-level radiotherapy before the POD10–14 neurological assessment, supporting the interpretation that these outcomes reflect early postoperative recovery patterns rather than postoperative radiotherapy response. Recovery was most strongly contextualized by residual preoperative motor function and ECOG performance status. These findings do not establish equivalence to earlier surgery or isolate the effect of surgery from multimodal oncologic care. Prospective studies incorporating standardized neurological assessment, radiotherapy sequencing, systemic therapy status, metastatic burden characterization, and longer functional follow-up are needed.

## Figures and Tables

**Table 1:** Baseline clinical, oncologic, radiological, disease-burden, and operative characteristics according to early neurological improvement.

Variable	Total (*N* = 41)	Neurological Improvement Yes (*n* = 20)	Neurological Improvement No (*n* = 21)	*p*
***Demographics***				
Age, years, median (IQR)	65.0 (58–71)	62.5 (58–69)	68.0 (61–74)	0.230
Male sex, *n* (%)	33 (80.5)	16 (80.0)	17 (81.0)	1.000
***Clinical and perioperative status***				
ECOG 0–2, *n* (%)	5 (12.2)	5 (25.0)	0 (0)	0.008
ECOG 3–4, *n* (%)	36 (87.8)	15 (75.0)	21 (100)	—
ASA class 2, *n* (%)	9 (22.0)	4 (20.0)	5 (23.8)	0.185
ASA class 3, *n* (%)	26 (63.4)	15 (75.0)	11 (52.4)	—
ASA class 4, *n* (%)	6 (14.6)	1 (5.0)	5 (23.8)	—
Emergency ASA modifier, *n* (%)	14 (34.1)	7 (35.0)	7 (33.3)	1.000
MSTFI 0–1, *n* (%)	13 (31.7)	7 (35.0)	6 (28.6)	0.744
MSTFI >1, *n* (%)	28 (68.3)	13 (65.0)	15 (71.4)	—
***Neurological and radiological status***				
Frankel A, *n* (%)	4 (9.8)	0 (0)	4 (19.0)	0.113
Frankel B, *n* (%)	14 (34.1)	7 (35.0)	7 (33.3)	—
Frankel C, *n* (%)	23 (56.1)	13 (65.0)	10 (47.6)	—
Bilsky grade 2, *n* (%)	10 (24.4)	4 (20.0)	6 (28.6)	0.719
Bilsky grade 3, *n* (%)	31 (75.6)	16 (80.0)	15 (71.4)	—
SINS 0–6 (stable), *n* (%)	6 (14.6)	3 (15.0)	3 (14.3)	0.380
SINS 7–12 (potentially unstable), *n* (%)	25 (61.0)	14 (70.0)	11 (52.4)	—
SINS 13–18 (unstable), *n* (%)	10 (24.4)	3 (15.0)	7 (33.3)	—
***Disease-burden and operative-target descriptors***				
Known primary tumor preoperatively, *n* (%)	20 (48.8)	13 (65.0)	7 (33.3)	0.063
Unknown primary tumor preoperatively, *n* (%)	21 (51.2)	7 (35.0)	14 (66.7)	—
Involved vertebral segments: 1, *n* (%)	5 (12.2)	2 (10.0)	3 (14.3)	0.836
Involved vertebral segments: 2, *n* (%)	6 (14.6)	3 (15.0)	3 (14.3)	—
Involved vertebral segments: 3, *n* (%)	8 (19.5)	5 (25.0)	3 (14.3)	—
Involved vertebral segments: >4, *n* (%)	22 (53.7)	10 (50.0)	12 (57.1)	—
Surgical target region: cervical, *n* (%)	3 (7.3)	1 (5.0)	2 (9.5)	0.797
Surgical target region: thoracic, *n* (%)	29 (70.7)	14 (70.0)	15 (71.4)	—
Surgical target region: lumbar, *n* (%)	9 (22.0)	5 (25.0)	4 (19.0)	—
SINS location category: junctional, *n* (%)	10 (24.4)	4 (20.0)	6 (28.6)	0.811
SINS location category: mobile, *n* (%)	8 (19.5)	4 (20.0)	4 (19.0)	—
SINS location category: semirigid, *n* (%)	23 (56.1)	12 (60.0)	11 (52.4)	—
***Tumor biology and laboratory values***				
Non-rapid-growth tumor, *n* (%)	27 (65.9)	16 (80.0)	11 (52.4)	0.100
Rapid-growth tumor, *n* (%)	14 (34.1)	4 (20.0)	10 (47.6)	—
Hemoglobin, g/dL, median (IQR)	12.1 (10–14)	12.2 (11–14)	11.7 (9–14)	0.179
Albumin, g/L, median (IQR)	35.0 (30–39)	35.0 (32–40)	31.0 (28–36)	0.042
Lymphocyte, ×10^9^/L, median (IQR)	1.4 (1.0–1.8)	1.5 (1.2–1.8)	1.4 (0.9–1.6)	0.361
Platelet, ×10^9^/L, median (IQR)	284 (226–344)	262 (226–302)	335 (232–362)	0.141
HALP score, median (IQR)	2.10 (1.25–2.73)	2.73 (1.48–3.59)	1.79 (0.88–2.23)	0.017
***Delay and surgical characteristics***				
Referral/access delay, *n* (%)	12 (29.3)	7 (35.0)	5 (23.8)	0.819
Patient decision/consent delay, *n* (%)	10 (24.4)	5 (25.0)	5 (23.8)	—
Oncologic sequencing/MDT delay, *n* (%)	8 (19.5)	3 (15.0)	5 (23.8)	—
Medical optimization delay, *n* (%)	5 (12.2)	2 (10.0)	3 (14.3)	—
Unknown/undocumented delay, *n* (%)	5 (12.2)	3 (15.0)	2 (9.5)	—
Intercurrent event delay, *n* (%)	1 (2.4)	0 (0)	1 (4.8)	—
Nonambulatory deficit, days, median (IQR)	7.0 (3–12)	7.5 (5–11)	7.0 (3–14)	0.753
Decompression alone, *n* (%)	13 (31.7)	6 (30.0)	7 (33.3)	0.808
Decompression + stabilization, *n* (%)	25 (61.0)	13 (65.0)	12 (57.1)	—
Corpectomy-based reconstruction, *n* (%)	3 (7.3)	1 (5.0)	2 (9.5)	—
Operative duration, min, median (IQR)	210 (170–270)	203 (175–270)	230 (170–275)	0.611
Intraoperative transfusion, *n* (%)	23 (56.1)	8 (40.0)	15 (71.4)	0.062

Data are *n* (%) for categorical variables and median (IQR) for continuous variables. Early neurological improvement was defined as an increase of at least one Frankel grade by postoperative days 10–14. Baseline Frankel grade was the grade documented immediately before surgery. *p*-values are from Mann–Whitney U tests for continuous variables, Fisher’s exact tests for two-category variables, and Pearson chi-square tests for multi-category variables. ASA, American Society of Anesthesiologists; ECOG, Eastern Cooperative Oncology Group; HALP, hemoglobin–albumin–lymphocyte–platelet; IQR, interquartile range; MDT, multidisciplinary team; MSTFI, Metastatic Spinal Tumor Frailty Index; SINS, Spinal Instability Neoplastic Score.

**Table 2:** Perioperative radiotherapy and systemic therapy context.

Variable	Total (*N* = 41)
***Preoperative radiotherapy***	
Preoperative index-level radiotherapy, *n* (%)	6 (14.6)
No preoperative index-level radiotherapy, *n* (%)	35 (85.4)
Any preoperative radiotherapy, *n* (%)	10 (24.4)
No documented preoperative radiotherapy, *n* (%)	31 (75.6)
***Postoperative radiotherapy***	
Postoperative index-level radiotherapy before POD10–14, *n* (%)	0 (0)
No postoperative index-level radiotherapy before POD10–14, *n* (%)	41 (100)
Any postoperative radiotherapy documented, *n* (%)	14 (34.1)
No postoperative radiotherapy documented, *n* (%)	12 (29.3)
Postoperative radiotherapy status unknown, *n* (%)	15 (36.6)
Radiotherapy field/dose/fractionation details available, *n* (%)	14 (34.1)
***Systemic therapy***	
Systemic therapy regimen documented, *n* (%)	19 (46.3)
No systemic therapy documented, *n* (%)	11 (26.8)
Systemic therapy status unknown, *n* (%)	11 (26.8)

Data are *n* (%). Index-level radiotherapy refers to treatment involving the surgically treated spinal level or maximal-compression level. Postoperative index-level radiotherapy before POD10–14 was specifically assessed because neurological outcomes were defined at POD10–14. RT, radiotherapy; POD, postoperative day.

**Table 3:** Multivariable Firth penalized logistic regression for early neurological improvement and early ambulation recovery.

Variable	Early Neurological Improvement Adjusted OR (95% CI)	*p*	Early Ambulation Recovery Adjusted OR (95% CI)	*p*
***Model A—Clinical predictors***				
Frankel C (vs. A–B)	1.270 (0.325–5.091)	0.730	6.205 (1.276–41.679)	0.023
ECOG 3–4 (vs. 0–2) ᵃ	0.049 (0.000–0.640)	0.018	0.046 (0.000–0.632)	0.017
Rapid-growth tumor (vs. non-rapid)	0.215 (0.036–0.916)	0.037	0.179 (0.016–1.048)	0.057
***Model B—Clinical predictors + HALP^z^***				
Frankel C (vs. A–B)	0.451 (0.064–2.355)	0.356	3.585 (0.614–26.712)	0.159
ECOG 3–4 (vs. 0–2) ᵃ	0.037 (0.000–0.492)	0.009	0.052 (0.000–0.598)	0.014
Rapid-growth tumor (vs. non-rapid)	0.206 (0.026–1.030)	0.055	0.253 (0.024–1.473)	0.132
HALP^z^ (per 1 SD increase)	6.292 (1.226–61.424)	0.017	1.902 (0.796–12.140)	0.198

Firth-penalized logistic regression with profile-likelihood confidence intervals. Early neurological improvement is ≥1 Frankel grade. Early ambulation recovery is achieving Frankel D/E. Model A included baseline Frankel grade, ECOG performance status, and tumor growth category. Model B added standardized HALP. Estimates are exploratory and hypothesis-generating because of the small sample size and limited event counts. ECOG, Eastern Cooperative Oncology Group; HALP, hemoglobin–albumin–lymphocyte–platelet; HALP^z^ = standardized HALP score; OR, odds ratio; CI, confidence interval; SD, standard deviation. ᵃ Extreme ORs for ECOG reflect Firth penalization under near-complete separation; direction and *p*-value are the clinically relevant outputs.

## Data Availability

The data presented in this study are available on request from the corresponding author due to patient confidentiality and institutional ethical restrictions. The data are not publicly available because they contain potentially identifiable retrospective clinical information.

## Supplementary Materials

The following supporting information can be downloaded at: https://www.mdpi.com/article/10.3390/curroncol33050299/s1, Table S1: Distribution of primary tumor subtypes across the adapted katagiri-style growth-category framework used in the analysis. Table S2: Baseline clinical, oncologic, radiological, disease-burden, and operative characteristics according to early ambulation recovery.
