# Supplementary material for: Early Neurological Improvement and Ambulation Recovery After Delayed Surgery in Surgically Selected Nonambulatory Metastatic Epidural Spinal Cord Compression: A Retrospective Cohort Study
Source: Curr Oncol. 2026 May 20;33(5):299. doi: 10.3390/curroncol33050299 (PMC13205622; doi:10.3390/curroncol33050299)
Supplement: Supplementary file 1 [file curroncol-33-00299-s001.zip › Supplementary Table S1.pdf]

**Supplementary Table S1.** Distribution of primary tumor subtypes across the adapted katagiri-style growth-category framework used in the analysis.

| **Growth Category** | **Primary Tumor Subtype** | **n** | **% of Cohort (N = 41)** |
|---|---|---|---|
| **Slow growth** | Lymphoma | 5 | 12.2 |
| | Multiple myeloma | 4 | 9.8 |
| | Prostate carcinoma | 5 | 12.2 |
| | Breast carcinoma | 2 | 4.9 |
| | **Subtotal, slow-growth tumors** | **16** | **39.0** |
| **Moderate growth** | Lung adenocarcinoma | 9 | 22.0 |
| | Renal cell carcinoma | 2 | 4.9 |
| | **Subtotal, moderate-growth tumors** | **11** | **26.8** |
| **Rapid growth** | Small-cell lung carcinoma | 3 | 7.3 |
| | High-grade neuroendocrine lung carcinoma | 1 | 2.4 |
| | Lung squamous / adenosquamous carcinoma | 2 | 4.9 |
| | Gastric carcinoma | 2 | 4.9 |
| | Colorectal carcinoma | 2 | 4.9 |
| | Pancreatic carcinoma | 3 | 7.3 |
| | Malignant melanoma | 1 | 2.4 |
| | **Subtotal, rapid-growth tumors** | **14** | **34.1** |
| | **Total** | **41** | **100.0** |
