# Supplementary material for: Early Neurological Improvement and Ambulation Recovery After Delayed Surgery in Surgically Selected Nonambulatory Metastatic Epidural Spinal Cord Compression: A Retrospective Cohort Study
Source: Curr Oncol. 2026 May 20;33(5):299. doi: 10.3390/curroncol33050299 (PMC13205622; doi:10.3390/curroncol33050299)
Supplement: Supplementary file 1 [file curroncol-33-00299-s001.zip › Supplementary Table S2.pdf]

Supplementary Table S2. Baseline clinical, oncologic, radiological, disease-burden, and operative characteristics according to early ambulation recovery.

Variable	Total (N = 41)	Ambulation Recovery Yes (n = 15)	Ambulation Recovery No (n = 26)	*p*
Demographics				
Age, years, median (IQR)	65.0 (58–71)	61.0 (49–69)	67.5 (61–74)	0.098
Male sex, n (%)	33 (80.5)	11 (73.3)	22 (84.6)	0.434
Clinical and perioperative status				
ECOG 0–2, n (%)	5 (12.2)	5 (33.3)	0 (0)	0.004
ECOG 3–4, n (%)	36 (87.8)	10 (66.7)	26 (100)	—
ASA class 2, n (%)	9 (22.0)	3 (20.0)	6 (23.1)	0.488
ASA class 3, n (%)	26 (63.4)	11 (73.3)	15 (57.7)	—
ASA class 4, n (%)	6 (14.6)	1 (6.7)	5 (19.2)	—
Emergency ASA modifier, n (%)	14 (34.1)	4 (26.7)	10 (38.5)	0.512
MSTFI 0–1, n (%)	13 (31.7)	6 (40.0)	7 (26.9)	0.492
MSTFI >1, n (%)	28 (68.3)	9 (60.0)	19 (73.1)	—
Neurological and radiological status				
Frankel A, n (%)	4 (9.8)	0 (0)	4 (15.4)	0.010
Frankel B, n (%)	14 (34.1)	2 (13.3)	12 (46.2)	—
Frankel C, n (%)	23 (56.1)	13 (86.7)	10 (38.5)	—
Bilsky grade 2, n (%)	10 (24.4)	2 (13.3)	8 (30.8)	0.277
Bilsky grade 3, n (%)	31 (75.6)	13 (86.7)	18 (69.2)	—
SINS 0–6 (stable), n (%)	6 (14.6)	3 (20.0)	3 (11.5)	0.720
SINS 7–12 (potentially unstable), n (%)	25 (61.0)	9 (60.0)	16 (61.5)	—
SINS 13–18 (unstable), n (%)	10 (24.4)	3 (20.0)	7 (26.9)	—
Disease-burden and operative-target descriptors				
Known primary tumor preoperatively, n (%)	20 (48.8)	9 (60.0)	11 (42.3)	0.341
Unknown primary tumor preoperatively, n (%)	21 (51.2)	6 (40.0)	15 (57.7)	—
Involved vertebral segments: 1, n (%)	5 (12.2)	2 (13.3)	3 (11.5)	0.643
Involved vertebral segments: 2, n (%)	6 (14.6)	1 (6.7)	5 (19.2)	—
Involved vertebral segments: 3, n (%)	8 (19.5)	4 (26.7)	4 (15.4)	—
Involved vertebral segments: >4, n (%)	22 (53.7)	8 (53.3)	14 (53.8)	—
Surgical target region: cervical, n (%)	3 (7.3)	1 (6.7)	2 (7.7)	0.408
Surgical target region: thoracic, n (%)	29 (70.7)	9 (60.0)	20 (76.9)	—
Surgical target region: lumbar, n (%)	9 (22.0)	5 (33.3)	4 (15.4)	—
SINS location category: junctional, n (%)	10 (24.4)	4 (26.7)	6 (23.1)	0.593
SINS location category: mobile, n (%)	8 (19.5)	4 (26.7)	4 (15.4)	—
SINS location category: semirigid, n (%)	23 (56.1)	7 (46.7)	16 (61.5)	—
Tumor biology and laboratory values				
Non-rapid-growth tumor, n (%)	27 (65.9)	12 (80.0)	15 (57.7)	0.186

Variable	Total (N = 41)	Ambulation Recovery Yes (n = 15)	Ambulation Recovery No (n = 26)	*p*
Rapid-growth tumor, n (%)	14 (34.1)	3 (20.0)	11 (42.3)	—
Hemoglobin, g/dL, median (IQR)	12.1 (10–14)	12.5 (11–14)	11.8 (9–14)	0.110
Albumin, g/L, median (IQR)	35.0 (30–39)	35.0 (32–40)	31.5 (29–37)	0.101
Lymphocyte, ×10⁹/L, median (IQR)	1.4 (1.0–1.8)	1.6 (1.3–1.9)	1.3 (0.9–1.6)	0.185
Platelet, ×10⁹/L, median (IQR)	284 (226–344)	270 (236–299)	312 (207–348)	0.330
HALP score, median (IQR)	2.10 (1.25–2.73)	3.03 (1.48–3.59)	1.82 (1.03–2.42)	0.026
Delay and surgical characteristics				
Referral/access delay, n (%)	12 (29.3)	6 (40.0)	6 (23.1)	0.607
Patient decision/consent delay, n (%)	10 (24.4)	4 (26.7)	6 (23.1)	—
Oncologic sequencing/MDT delay, n (%)	8 (19.5)	1 (6.7)	7 (26.9)	—
Medical optimization delay, n (%)	5 (12.2)	2 (13.3)	3 (11.5)	—
Unknown/undocumented delay, n (%)	5 (12.2)	2 (13.3)	3 (11.5)	—
Intercurrent event delay, n (%)	1 (2.4)	0 (0)	1 (3.8)	—
Nonambulatory deficit, days, median (IQR)	7.0 (3–12)	8.0 (5–13)	7.0 (3–12)	0.414
Decompression alone, n (%)	13 (31.7)	6 (40.0)	7 (26.9)	0.686
Decompression + stabilization, n (%)	25 (61.0)	8 (53.3)	17 (65.4)	—
Corpectomy-based reconstruction, n (%)	3 (7.3)	1 (6.7)	2 (7.7)	—
Operative duration, min, median (IQR)	210 (170–270)	200 (175–270)	228 (150–275)	0.839
Intraoperative transfusion, n (%)	23 (56.1)	6 (40.0)	17 (65.4)	0.191

Data are n (%) for categorical variables and median (IQR) for continuous variables. Early ambulation recovery was defined as postoperative Frankel grade D or E by postoperative days 10–14. Baseline Frankel grade was the grade documented immediately before surgery. p-values are from Mann–Whitney U tests for continuous variables, Fisher's exact tests for two-category variables, and Pearson chi-square tests for multi-category variables. ASA, American Society of Anesthesiologists; ECOG, Eastern Cooperative Oncology Group; HALP, hemoglobin–albumin–lymphocyte–platelet; IQR, interquartile range; MDT, multidisciplinary team; MSTFI, Metastatic Spinal Tumor Frailty Index; SINS, Spinal Instability Neoplastic Score.
